# Familial non-inflammatory, non-atherosclerotic vasculopathy presented with acute coronary syndrome

**DOI:** 10.1093/ehjimp/qyaf039

**Published:** 2025-04-03

**Authors:** Hikaru Masuda, Riku Arai, Yuki Nakajima, Yutaka Koyama, Nobuhiro Murata, Yasuo Okumura, Masashi Tanaka, Hiroyuki Hao

**Affiliations:** Division of Cardiology, Department of Medicine, Nihon University School of Medicine, 30-1 Oyaguchi-Kamimachi, Itabashi-Ku, Tokyo 173-8610, Japan; Division of Cardiology, Department of Medicine, Nihon University School of Medicine, 30-1 Oyaguchi-Kamimachi, Itabashi-Ku, Tokyo 173-8610, Japan; Division of Cardiology, Department of Medicine, Nihon University School of Medicine, 30-1 Oyaguchi-Kamimachi, Itabashi-Ku, Tokyo 173-8610, Japan; Division of Human Pathology, Department of Pathology and Microbiology, Nihon University School of Medicine, Tokyo, Japan; Division of Cardiology, Department of Medicine, Nihon University School of Medicine, 30-1 Oyaguchi-Kamimachi, Itabashi-Ku, Tokyo 173-8610, Japan; Division of Cardiology, Department of Medicine, Nihon University School of Medicine, 30-1 Oyaguchi-Kamimachi, Itabashi-Ku, Tokyo 173-8610, Japan; Department of Cardiovascular Surgery, Nihon University School of Medicine, Tokyo, Japan; Division of Human Pathology, Department of Pathology and Microbiology, Nihon University School of Medicine, Tokyo, Japan

**Keywords:** acute coronary syndrome, fibromuscular dysplasia, arteriosclerosis, pathological diagnosis, intensive care, multi-modality imaging

A 17-year-old male presented to our hospital after experiencing transient loss of consciousness during exercise. Elevated cardiac markers and ST-segment changes on the electrocardiogram led to a diagnosis of non-ST elevation myocardial infarction (*Figure 1*, A). His family history revealed multiple sudden deaths due to cardiovascular causes (*Figure 1*, B). His renal, hepatic, and lipidic profiles were within normal limits. Enhanced whole-trunk computed tomography and magnetic resonance angiography revealed multiple vascular stenoses in brachiocephalic artery *(Figure 1*, C1), aortic arch (*Figure 1*, C2), descending aorta (*Figure 1*, C3), abdominal aorta (*Figure 1*, C4), left pulmonary artery (*Figure 1*, C5), left internal thoracic artery (LITA) (*Figure 1*, D), mid-right coronary artery (*Figure 1*, E1), left main trunk, proximal and mid-left anterior descending artery (*Figure 1*, E2, E3), and bilateral internal carotid arteries (*Figure 1*, F). Blood tests showed no evidence of inflammatory, collagen, coagulation or malignant disorders and genetic tests account for hereditary conditions such as PHACTR1, RNF213 were negative, so we ruled out active inflammatory or thrombotic diseases. Coronary artery bypass graft surgery was performed using the left and right internal thoracic arteries as free grafts, along with saphenous vein grafts. The stenotic lesion of the LITA was resected and submitted for pathological examination, while a healthy segment was used as the graft. Warfarin and aspirin were prescribed for post-operative medication. Pathological examination of LITA revealed non-inflammatory, non-atherosclerotic, and non-thrombotic lesions characterized by collagen and smooth muscle cell proliferation, suggesting intimal fibromuscular dysplasia (FMD) in Hematoxylin–Eosin stain (*Figure 1*, G1, G2) and Masson trichrome stain (*Figure 1*, G3).

**Figure 1:**
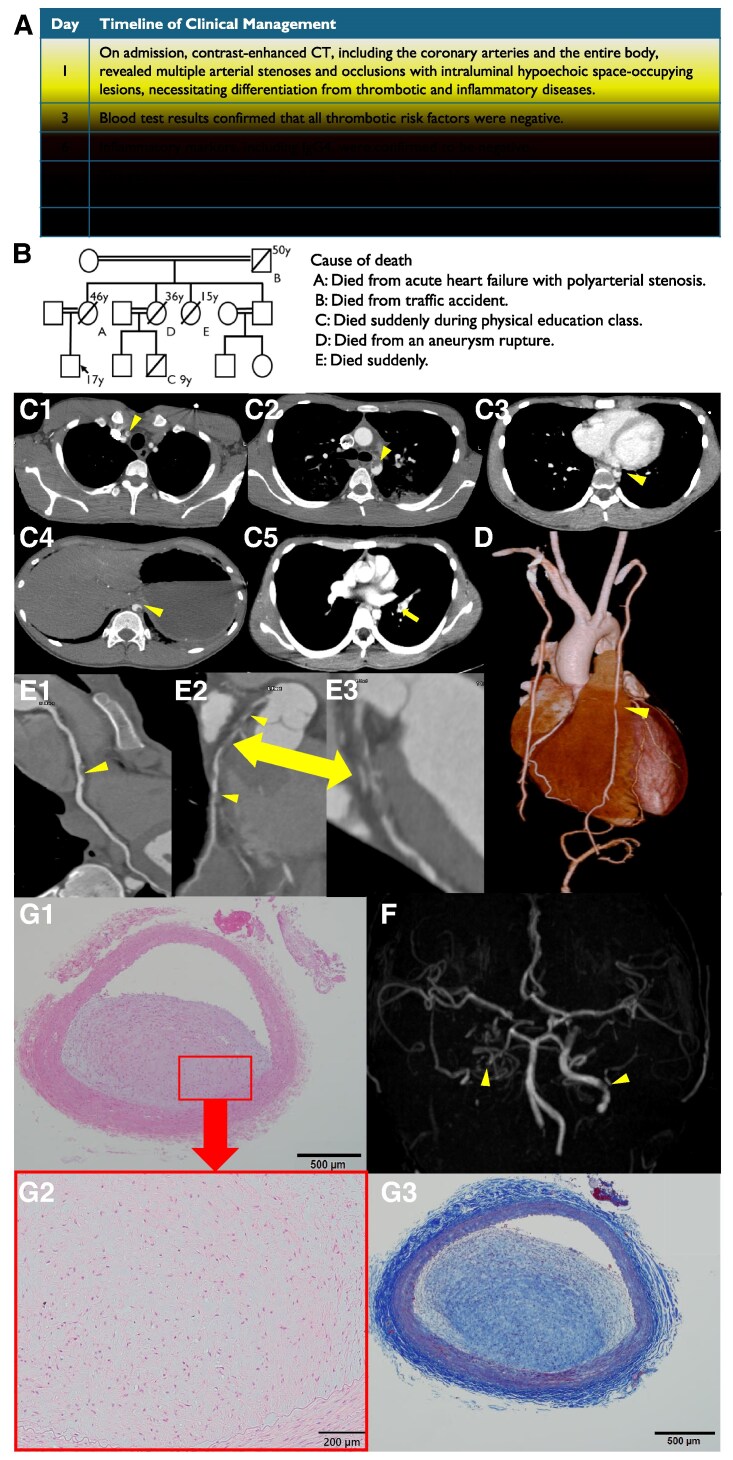
After excluding thrombotic and inflammatory diseases, coronary artery bypass graft was performed on day 8, and the patient was discharged without complications on day 24 (*A*). His family tree revealed five deaths within fourth-degree family members (*B*). Enhanced computed tomography showed multiple low-density lesions protruding into the vascular lumen in brachiocephalic artery (*C1*), aortic arch (*C2*), descending aorta (*C3*), abdominal aorta (*C4*), and left pulmonary artery (*C5*). Polyvascular stenoses involving the left internal thoracic artery (*D*), significant stenosis in the mid right coronary artery (*E1*), the left main trunk, proximal left anterior descending artery (LAD), and total occlusion in the mid LAD (*E2*) with positive remodeling (*E3*, yellow arrow) were observed on computed tomography angiography. Additionally, magnetic resonance angiography revealed stenoses in the bilateral internal carotid arteries (*F*). The superior portion of the stenotic lesion was excised during the operation, and histological specimens were prepared. The obstructive lesion was characterized by proliferation of collagen fibers and smooth muscle cells, as demonstrated by hematoxylin-eosin stain (*G1*, *G2*) and Masson's trichrome stain (*G3*).

This case was ultimately diagnosed as acute myocardial infarction caused by the intimal FMD.

## Data Availability

No additional data were created or analysed for the purposes of this study.

## Lead author biography

Hikaru Masuda graduated from Nihon University School of Medical in 2014. He is currently working as a senior resident in the Department of Cardiology of Nihon University School of Medicine, Itabashi Hospital.

**Consent:** This patient provided informed consent for this paper in our hospital.

